# Loss of amphiregulin reduces myoepithelial cell coverage of mammary ducts and alters breast tumor growth

**DOI:** 10.1186/s13058-018-1057-0

**Published:** 2018-10-26

**Authors:** Serena P. H. Mao, Minji Park, Ramon M. Cabrera, John R. Christin, George S. Karagiannis, Maja H. Oktay, Dietmar M. W. Zaiss, Scott I. Abrams, Wenjun Guo, John S. Condeelis, Paraic A. Kenny, Jeffrey E. Segall

**Affiliations:** 1 Department of Anatomy and Structural Biology, Albert Einstein College of Medicine, 1301 Morris Park Avenue, Bronx, NY 10461 USA; 2 Department of Cell Biology, Albert Einstein College of Medicine, Bronx, NY 10461 USA; 3 Gruss Lipper Biophotonics Center, Albert Einstein College of Medicine, Bronx, NY 10461 USA; 4 Integrated Imaging Program, Albert Einstein College of Medicine, Bronx, NY 10461 USA; 5 Institute of Immunology and Infection Research, University of Edinburgh, Edinburgh, UK; 6 Department of Immunology, Roswell Park Comprehensive Cancer Center, Buffalo, NY 14263 USA; 7 Kabara Cancer Research Institute, Gundersen Medical Foundation, La Crosse, WI 54601 USA

**Keywords:** Amphiregulin, Mammary ductal development, MMTV-PyMT, Breast cancer

## Abstract

**Background:**

Amphiregulin (AREG), a ligand of the epidermal growth factor receptor, is not only essential for proper mammary ductal development, but also associated with breast cancer proliferation and growth. In the absence of AREG, mammary ductal growth is stunted and fails to expand. Furthermore, suppression of AREG expression in estrogen receptor-positive breast tumor cells inhibits in-vitro and in-vivo growth.

**Methods:**

We crossed AREG-null (AREG^−/−^) mice with the murine luminal B breast cancer model, MMTV-PyMT (PyMT), to generate spontaneous breast tumors that lack AREG (AREG^−/−^ PyMT). We evaluated tumor growth, cytokeratin-8 (K8)-positive luminal cells, cytokeratin-14 (K14)-positive myoepithelial cells, and expression of AREG, Ki67, and PyMT. Primary myoepithelial cells from nontumor-bearing AREG^+/+^ mice underwent fluorescence-activated cell sorting and were adapted to culture for in-vitro coculture studies with AT-3 cells, a cell line derived from C57Bl/6 PyMT mammary tumors.

**Results:**

Intriguingly, PyMT-induced lesions progress more rapidly in AREG^−/−^ mice than in AREG^+/+^ mice. Quantification of K8^+^ luminal and K14^+^ myoepithelial cells in non-PyMT AREG^−/−^ mammary glands showed fewer K14^+^ cells and a thinner myoepithelial layer. Study of AT-3 cells indicated that coculture with myoepithelial cells or exposure to AREG, epidermal growth factor, or basic fibroblast growth factor can suppress PyMT expression. Late-stage AREG^−/−^ PyMT tumors are significantly less solid in structure, with more areas of papillary and cystic growth. Papillary areas appear to be both less proliferative and less necrotic. In The Cancer Genome Atlas database, luminal-B invasive papillary carcinomas have lower AREG expression than luminal B invasive ductal carcinomas.

**Conclusions:**

Our study has revealed a previously unknown role of AREG in myoepithelial cell development and PyMT expression. AREG expression is essential for proper myoepithelial coverage of mammary ducts. Both AREG and myoepithelial cells can suppress PyMT expression. We find that lower AREG expression is associated with invasive papillary breast cancer in both the MMTV-PyMT model and human breast cancer.

**Electronic supplementary material:**

The online version of this article (10.1186/s13058-018-1057-0) contains supplementary material, which is available to authorized users.

## Background

Breast cancer remains the most common form of cancer among women in the USA. In the most recent estimates, there are over 250,000 new cases and 40,000 new deaths predicted in 2018 alone [1]. Overexpression of epidermal growth factor receptor (EGFR) has been shown to be an important predictor of early recurrence and death in breast cancer [2, 3]. Historically, patients with positive EGFR status were associated with shorter relapse-free and overall survival. However, therapies targeting EGFR in breast cancer have been met with many challenges and little success [4–6]. Amphiregulin (AREG), a ligand of EGFR, has been found to be overexpressed in estrogen receptor (ER)-positive breast cancer [7]. Further evidence shows that loss of AREG in breast cancer cells can stunt tumor proliferation, growth, and invasiveness in vitro and in vivo [8–10]. In addition to breast cancer, AREG has been shown to play an important role in mammary gland development. During puberty, AREG is the only EGFR ligand that is transcriptionally activated by estrogen receptor signaling in the mammary gland [11]. In the absence of AREG, the mammary ductal tree fails to expand and remains as a rudimentary tree throughout adulthood. In mammary gland transplant studies, epithelial AREG expression and stromal EGFR expression have been identified as critical for proper mammary gland development [12]. Interestingly, when AREG-null epithelial cells are transplanted into a cleared mammary gland, regardless of EGFR status in the stroma, the resultant gland shows a lack of cytokeratin-14 (K14) protein, a marker for myoepithelial cells [13]. While it is unknown whether AREG supports development and maintenance of myoepithelial cells, some evidence suggests that under low EGFR signaling conditions, mammary stem cells (MaSCs) preferentially differentiate into luminal, not myoepithelial, cells [14]. In the same study, it was shown that in the presence of AREG, but not EGF, normal ductal development occurred. Therefore, it is possible that AREG is not only important for the expansion of the ductal tree, but also for proper differentiation of epithelial progenitor cells into luminal and myoepithelial cells.

Little is known about how AREG expression alters breast cancer initiation and progression. In our studies, we sought to better understand the role of AREG in breast cancer using the MMTV-PyMT (PyMT) mouse model. The PyMT model is a widely used murine model of breast cancer due to its similarities in tumor progression stages to human breast cancer [15, 16]. Furthermore, activation of PyMT drives many oncogenic pathways involving key signaling molecules, such as Src, Ras, and PI3K that are overexpressed in many different human cancers [17–19]. By crossing PyMT mice with AREG-null mice, we have evaluated the properties of the spontaneous PyMT breast tumor model in the absence of AREG.

In the studies described, we show for the first time novel functions of AREG in mammary gland development, PyMT expression, and breast cancer growth.

## Methods

### Mice

All animal studies were conducted with approval by the Albert Einstein College of Medicine Institutional Animal Care and Use Committee (IACUC). All husbandry was provided by the Institute of Animal Studies (IAS) under the supervision of veterinarians at the institution. Mice were maintained in a pathogen-free facility under controlled light cycles and temperatures. In our animal experiments, we used transgenic mice expressing the polyoma middle-T antigen (PyMT) controlled by the mammary tumor virus (MMTV) in the C57Bl/6 background as our murine breast cancer model. These animals were provided by Dr Jeffrey W. Pollard at our institution, bred in-house, and maintained on the C57Bl/6 background. To explore the role of amphiregulin (AREG) in breast cancer, we used AREG-knockout (AREG^−/−^) mice in the same background [20]. Genotypes of offspring were identified by quantitative PCR (qPCR) via Transnetyx (Cordova, TN, USA).

### Lesion growth and histological measurements

Palpable lesion growth was evaluated three times weekly using a digital caliper. Animals were sacrificed once the largest lesion reached 1 cm in diameter. Animals whose lesions ruptured prior to reaching the appropriate size were excluded from our analyses. AREG^−/−^ lesions had a greater tendency to be cystic and may have ruptured more easily than AREG^+/+^ lesions as they grew bigger. Lesions were excised and fixed in 10% formalin for 72 h. Tissues were then embedded in paraffin and serially sectioned for immunohistochemistry and immunofluorescence studies. Tumor progression was evaluated by a breast cancer pathologist (MHO) for presence of hyperplasia, ductal carcinoma in situ, and adenocarcinoma in a blinded fashion. All IHC and H&E staining was performed by the Histology and Comparative Pathology core facility at Albert Einstein College of Medicine. Slides were scanned using the 3DHISTECH Pannoramic 250 flash II digital whole slide scanner. Cystic evaluation was completed by examining 1-cm lesions for the presence of cysts. If a lesion had at least one cyst, it was considered cystic in our analysis.

### Necrosis analysis

H&E stains of 1-cm AREG^+/+^ PyMT (*N* = 32) and AREG^−/−^ PyMT (*N* = 22) tumors were evaluated for the presence of necrosis. Quantification of the percentage of necrosis per tumor was determined by averaging the percentage of necrosis in individual 5× fields. The fields used in the analysis were determined randomly using a grid placed over the tissue image in Pannoramic Viewer so as to select unbiased areas. Solid and papillary areas were analyzed separately to determine the amount of necrosis in each type of histological structure. Statistical analysis was performed using the Mann–Whitney test.

### Circulating tumor cell measurement

The in-vivo intravasation assay for circulating tumor cells (CTCs) was performed as described previously [21–24]. A 25-gauge needle and syringe coated with heparin was inserted into the right ventricle of the heart of anesthetized mice and up to 1 ml of blood was collected from the heart puncture and transferred to a 15-ml tube with 10 ml of 1× RBC lysis buffer (cat. 00-4300-54; Affymetrix). After a 10-min incubation at room temperature, the cell suspension was pelleted by centrifugation at 200 x g for 5 min. The cell pellet was reconstituted in 10 ml of Dulbecco’s modified Eagle medium DMEM/F12 (cat. 11320–033; Gibco), supplemented with 20% fetal bovine serum (FBS)-premium select (cat. S11510; Atlanta Biologicals) and plated in a 10-cm tissue culture-treated Petri dish. Media were changed after 48 h. After a 1-week incubation, single tumor cells attached on the dish were counted. Finally, the cell count was normalized to 1 ml of blood.

### Metastasis measurements

After mice were sacrificed, the lungs were inflated with 10% formalin and fixed for 72 h. After fixation, the samples were embedded in paraffin and sectioned. Lungs were stained with hematoxylin and eosin (H&E). The number of metastatic foci was counted and their area was measured.

### Carmine staining

The mammary fat pads were evaluated using carmine staining as described previously [25]. Briefly, glands were fixed in Carnoy’s fixative overnight at 4 °C. Glands were rehydrated in serial dilutions of ethanol and rinsed once with water followed by staining with 0.2% carmine alum solution overnight at room temperature. The next day, glands were incubated in 1% HCl/70% EtOH solution for 4 h to remove the excess carmine stain. Glands were then dehydrated in increasing concentrations of ethanol. A 1-h xylene incubation was used to clear the tissue. Cleared glands were mounted in Permount (cat. SP15–500; Fisher Scientific). Finally, the slides were scanned using a conventional digital scanner.

### Immunofluorescence

Slides were deparaffinized and stained as described previously [24]. The following primary antibodies were used: PyMT (cat. NB100-2749; Novus Biologicals), IBA1 (cat. NB100-1028; Novus Biologicals), CD31 (cat. 77699; Cell Signaling), KRT8 (cat. TROMA-I; Developmental Studies Hybridoma Bank), and KRT14 (cat. 905304; Biolegend). After deparaffinization, slides were placed in a 1× target retrieval solution (cat. S169984-2; Agilent Technologies) and incubated overnight in the Retriever 220 V (cat. 62700-20; Electron Microscopy Sciences) for antigen retrieval. Slides were washed in 1× PBS and incubated with blocking buffer (10% donkey serum/0.1% Triton-X100) for 1 h at 4 °C. Primary antibodies were diluted in 1× PBS-T at the following concentrations: PyMT 1:100, IBA1 1:100, CD31 1:250, KRT8 1:30, and KRT14 1:1000. Samples were incubated with primary antibody solutions overnight at 4 °C. Before secondary antibody incubation, slides were washed three times in 1× PBS-T for 5 min each. The secondary antibodies used were Alexa Fluor 647 donkey anti-rabbit IgG (cat. A31573; Life Technologies), Alexa Fluor 488 donkey anti-Rat IgG (cat. A21208; Life Technologies), and Alexa Fluor 568 donkey anti-goat (cat. A11077; Life Technologies). Secondary antibodies were diluted in 1× PBS-T at 1:250. Secondary antibody incubation was performed at room temperature for 1 h. Slides were mounted using Dapi-Fluoromount-G (cat. OB010020; Southern Biotech) and stored at 4 °C. Slides were scanned using the 3DHISTECH Pannoramic 250 flash II digital whole slide scanner. The 20 × 0.8 NA objective lens was used for all scans.

### PyMT expression quantitation and analysis

Three to five fields per sample were chosen for analysis. Images were taken in Pannoramic Viewer and opened in ImageJ. All images were converted to 8-bit and the same threshold was applied to all images. After the threshold was designated, the region of interest (ROI) covered only the mammary ducts or lesions. The surrounding vessels, fat, and stroma were excluded. The PyMT immunofluorescence intensity was analyzed only within the ROIs.

### In-situ hybridization

In-situ hybridization experiments were performed using the manufacturer’s protocol for the BaseScope™ Assay (cat. 322971; ACD). After the signal was detected, the slides were blocked with 4% donkey serum (cat. D9663-10ML; Sigma-Aldrich) in 0.1% 1× PBS-T for 1 h at room temperature. Slides were stained for PyMT using the protocol described earlier.

### Mammary epithelial cell counting and myoepithelial cell layer thickness measurement

Mammary ducts were immunostained for K8 and K14 to visualize luminal (K8^+^) and myoepithelial (K14^+^) cells. The ratio of myoepithelial cells to total mammary epithelial cells was counted manually and calculated for five ducts per mammary fat pad. At least three AREG^+/+^ mice and three AREG^−/−^ mice were used for this analysis. In cases of uncertainty, a confocal microscope was utilized to differentiate the different cell layers. The outlines of myoepithelial cells were traced to calculate the thickness of the myoepithelial cells.

### Mammary epithelial cell isolation

Methods used to retrieve mammary epithelial cells (MECs) from mice were described previously [26]. Excised mammary glands were placed in ice-cold PBS. Glands were finely minced on a bacterial Petri dish and resuspended in 3 ml/mouse DMEM/F12 (cat. 11320-033; Gibco). Then 300 units/ml collagenase III (cat. LS004182; Worthington), 50 μg/ml DNase I (cat. LS002139; Worthington), and 5 μM Y-27632 (cat. Y-5301; LC Labs) were added and incubated at 37 °C for 2 h under constant rotation. Afterward, the digestion mixture was thoroughly mixed and PBS was added to 15 ml. The mixture was centrifuged at 300 × *g* for 5 min. To remove the erythrocytes, the cell pellet was resuspended with 1 ml RBC lysis buffer (8.3 g/L ammonium acetate, 10 mM Tris–HCl pH 7.5) and incubated on ice for 1 min. The cell mixture was thoroughly mixed, PBS was added to 15 ml, and the mixture was centrifuged again. The cell pellet was resuspended in 1 ml 0.05% Trypsin–EDTA (cat. MT25052CI; Corning) and incubated at 37 °C for 5 min. Trypsin was diluted with 10% FBS in DMEM/F12 and the cell mixture was centrifuged. To dissociate the luminal and myoepithelial cells, the cell pellet was resuspended in 1 ml DMEM/F12, 1 U/ml Dispase (cat. LS02109; Worthington), and 100 μg/ml DNase. The cell mixture was incubated at 37 °C for 5 min and passed through a 40-μm cell strainer. Then 5 ml of PBS was added to the final cell suspension. The cell number was determined using a hemocytometer. The cells were centrifuged and resuspended in FACS buffer (1 ml FBS, 31 ml PBS, 8 ml 10 mM EDTA) at 1 million cells/100 μl.

### Fluorescence-activated cell sorting

To isolate myoepithelial cells from the cell suspension, the cells were labeled with 1:100 biotin TER-119 (cat. 116204; Biolegend), biotin CD45 (cat. 103104; Biolegend), biotin CD31 (cat. 102404; Biolegend), APC EpCAM (cat. 17–5791-80; Affymetrix), and PerCP-Cy5.5 CD49f (cat. 562475; BD Biosciences). After a 15-min incubation on ice, streptavidin v450 (cat. 560797; BD Biosciences) and 1 μg/ml DAPI (cat. 422801; Biolegend) were added for another 15-min incubation. Cells were washed once and resuspended in fluorescence-activated cell sorting (FACS) buffer. The lineage-negative (TER-119^−^CD45^−^CD31^−^) EpCAM^−^CD49f^+^ cells were identified as myoepithelial cells.

### Cell lines and cell culture

Sorted myoepithelial cells were centrifuged and resuspended in 1:20 Matrigel (cat. 354234; Corning) and cultured in advanced-DMEM/F12 (cat. 12634010; Life Technologies) supplemented with 10 ng/ml EGF (cat. 585506; Biolegend), 20 ng/ml bFGF (cat. 710304; Biolegend), 4 μg/ml heparin (cat. H3149-10KU; Sigma-Aldrich), 5% newborn calf serum (cat. SH3011803; HyClone), and 5 μM Y-27632.

AT-3 cells, a murine breast cancer cell line derived from MMTV-PyMT tumors in the C57Bl/6 background, were cultured at 7% CO_2_ in DMEM high glucose (cat. MT-10-013-CV; Corning) supplemented with 10% FBS premium-select, penicillin–streptomycin (cat. MT30002CI; Corning), 15 mM HEPES (cat. 15630080; Life Technologies), 2 mM l-glutamine (cat. SH3003401; HyClone), NEAA (cat. SH3023801; HyClone), 1 mM sodium pyruvate (cat. 13-115E; Lonza Walkersville), and 1:250,000 2-mercaptoethanol (cat. M6250-100ML; Sigma Aldrich).

### In-vitro experiments

For the coculture experiments, 300,000 primary myoepithelial cells and 300,000 AT-3 cells were plated together in a six-well tissue culture plate overnight. In the control well, 300,000 AT-3 cells were plated. Cells were lysed on the following day using Buffer RLT Plus (cat. 1053393; Qiagen) and RNA was extracted using the RNeasy Plus Mini Kit (cat. 74134; Qiagen). Subsequently, cDNA was synthesized and amplified using the Superscript II system (cat. 11904-018; Thermofisher Scientific).

For the stimulation experiments, 300,000 AT-3 cells were plated overnight. On the following day, the media were switched to those containing either 10 ng/ml EGF, 10 ng/ml bFGF, 100 ng/ml AREG (cat. 989-AR-100; R&D Systems), or both EGF and bFGF. Cells were lysed after a 24-h incubation period.

### Quantitative RT-PCR

The gene expression level of PyMT was measured in the coculture and stimulation experiments using a SYBR Green Real-Time Master Mix and PyMT primers. The PyMT primer sequences were TTCGATCCGATCCTAGATGC and TGCCGGGAACGTTTTATTAG. PyMT expression was normalized to GAPDH expression. The GAPDH primer sequences were CTGGAGAAACCTGCCAAGTA and TGTTGCTGTAGCCGTATTCA. Each experiment was done in triplicate and repeated at least three independent times. Relative PyMT expression levels were derived from the GAPDH mean cycle threshold (Ct) values subtracted by the PyMT Ct values. Myoepithelial cells and AT-3 cells had similar levels of GAPDH. In coculture experiments, ΔCt values were adjusted to compensate for a twofold dilution in PyMT expression level. Changes in relative PyMT expression levels between experiment and control were measured as the fold change (ΔΔCt).

### TCGA analysis

The Cancer Genome Atlas (TCGA) Research Network (http://cancergenome.nih.gov/) provided a database of human breast cancer patient data which we analyzed for AREG expression and histological subtype. Since the MMTV-PyMT model was characterized as most similar to the luminal B subtype in human breast cancer, we chose our sample population from patient tumors that were identified as luminal B subtype. With the final sample of 123 patient samples, 115 were nonpapillary invasive ductal cancer (IDC) and eight were invasive papillary breast cancer (IPC). AREG RNAseq expression data provided by TCGA for these patient samples were then evaluated [27, 28].

### Statistical analyses

All statistical analyses were carried out using GraphPad Prism 7 software. Statistical analyses were performed using tests as indicated in the figure legends.

## Results

### Expansion and progression of tumorigenic lesions is accelerated in the absence of AREG

**Fig. 1:**
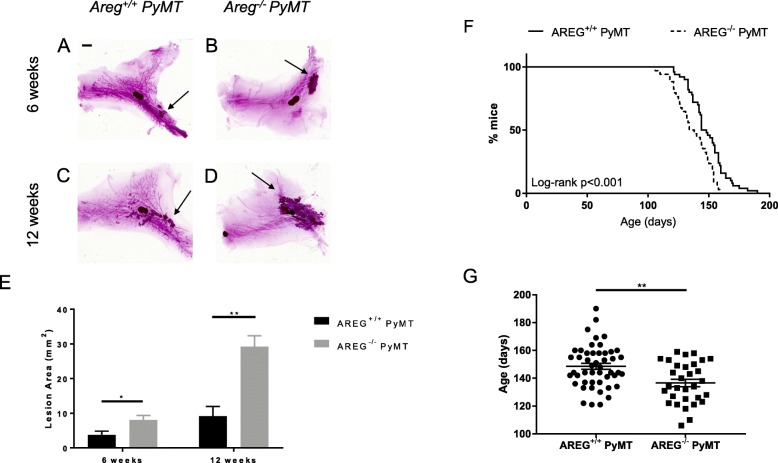
In absence of AREG, growth of lesions accelerated. **a–d** Representative images of carmine-stained AREG^+/+^ PyMT and AREG^−/−^ PyMT mammary fat pads at 6 weeks and 12 weeks. Lesions marked by arrows. Scale bar shows 2000 μm. **e** Total lesion area measured for AREG^+/+^ PyMT (black bars) and AREG^−/−^ PyMT (gray bars) mice, respectively. At least seven animals measured for 6-week group and at least three animals for 12-week group. **f**, **g** AREG^−/−^ PyMT palpable lesions reached 1-cm diameter more rapidly. **f** Kaplan–Meier plot of percentage of mice with palpable lesions less than 1 cm for AREG^−/−^ PyMT mice (dotted line, *N* = 33) and AREG^+/+^ PyMT mice (solid line, *N* = 50). **g** Mean and SEM of data in (**f**). Statistical analysis performed using a log-rank test (**f**) and *t* test (**e**, **g**). *p < 0.05, **p < 0.01. AREG amphiregulin, PYMT polyoma middle-T antigen

We examined the role of AREG in breast cancer using the MMTV-PyMT (PyMT) model in AREG^−/−^ mice. The appearance of lesions by carmine staining was visible in the mammary fat pads (MFPs) of both AREG^+/+^ PyMT (Fig. 1a) and AREG^−/−^ PyMT (Fig. 1b) females as early as 6 weeks of age. Lesions were larger in AREG^−/−^ PyMT mice at 6 weeks, and by 12 weeks the difference in size of the lesions was even more dramatic (Fig. 1c–e). Interestingly, the lesions in AREG^+/+^ PyMT mice were found in distinct regions in the ductal tree while in AREG^−/−^ PyMT mice much of the ductal tree appeared to convert into the growing lesion. The appearance of multiple lesions in the AREG^+/+^ PyMT ductal tree is consistent with previous reports [16]. We measured the growth of palpable lesions in AREG^+/+^ PyMT and AREG^−/−^ PyMT mice three times per week using a digital caliper until the largest palpable lesion reached a diameter of 1 cm (Additional file 1: Figure S1). The largest lesion reached the 1-cm endpoint significantly faster in AREG^−/−^ PyMT mice (Fig. 1f). The average ages at which AREG^+/+^ PyMT mice and AREG^−/−^ PyMT mice reached the endpoint were 144 days and 134 days, respectively (Fig. 1g).

**Fig. 2:**
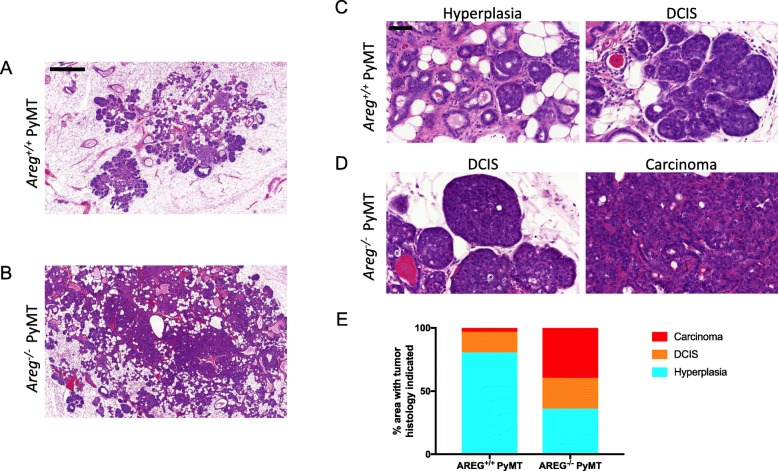
Loss of AREG enhances progression to invasive carcinoma. Progression of lesions evaluated based on stages: hyperplasia, ductal carcinoma in situ (DCIS), invasive carcinoma. **a**, **b** Low-magnification representative H&E images of MFPs of 12-week-old AREG^+/+^ PyMT (**a**) and AREG^−/−^ PyMT (**b**) mice. Scale bar shows 500 μm. **c**, **d** High-magnification images of stages of progression as seen in AREG^+/+^ PyMT (**c**) and AREG^−/−^ PyMT (**d**) mice. Scale bar shows 50 μm. **e** Greater proportion of AREG^−/−^ lesions identified as invasive carcinomas while most AREG^+/+^ lesions were hyperplastic. *N* = 10. AREG amphiregulin, PYMT polyoma middle-T antigen

Consistent with the growth data, tumor progression was also accelerated in the AREG^−/−^ PyMT mice. Histology of the lesions was evaluated by a breast cancer pathologist (MHO). Based on these assessments, the lesions of 12-week-old AREG^+/+^ PyMT mice were found to be predominantly at the stage of hyperplasia, with some limited areas of DCIS and invasive carcinoma (Fig. 2a, c). However, lesions of age-matched AREG^−/−^ PyMT mice showed more areas that had progressed to DCIS and invasive carcinoma (Fig. 2b, d, e).

To determine whether AREG-associated effects in tumor growth and progression were associated with changes in intravasation and metastasis, we examined metastases in the lungs as well as circulating tumor cells (CTCs) in these animals. Overall, we found no difference in metastasis as measured by the number of foci or total area in the lungs (Additional file 2: Figure S2A, B) or number of CTCs (Additional file 2: Figure S2C). In summary, loss of AREG appears to enhance expansion of tumorigenic lesions and accelerate tumor progression, but does not have an effect on intravasation and metastasis.

**Fig. 3:**
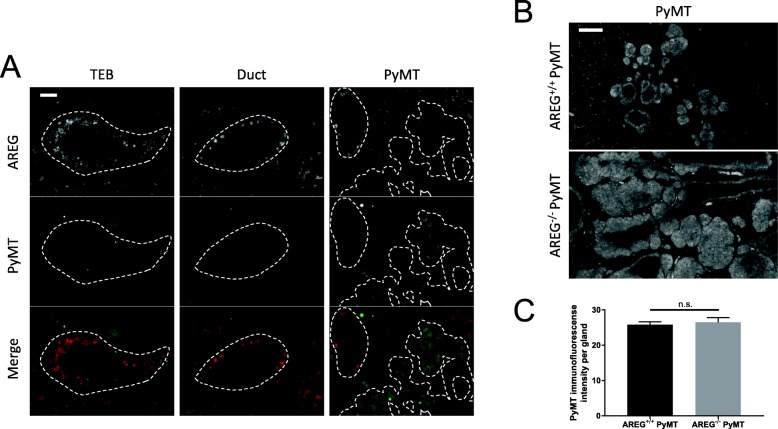
AREG not expressed in PyMT lesions. **a** Tissue sections hybridized in situ with AREG probe (dots in ducts), and PyMT protein detected by immunofluorescence. Individual channels shown in gray scale; merged image shows AREG in red and PyMT in green. Ductal structures or lesions outlined in white, and labeling of surrounding adipose tissue is nonspecific background staining. Scale bar shows 50 μm. **b** Representative images of PyMT immunofluorescent staining of 6-week-old AREG^+/+^ PyMT and AREG^−/−^ PyMT MFPs, respectively. Scale bar shows 100 μm. **c** At least 10 mammary glands were analyzed for PyMT staining intensity. Statistical analysis performed using *t* test. AREG amphiregulin, n.s. not significant, PYMT polyoma middle-T antigen, TEB terminal end bud

Using in-situ hybridization, we confirmed that AREG is expressed in AREG^+/+^ animals in the ductal cells and TEBs, consistent with previous studies (Fig. 3a) [13], with no expression in AREG^−/−^ animals. We then compared the localization of AREG and PyMT expression in AREG^+/+^ PyMT animals and found that they were inversely related. PyMT expression was absent in ducts and TEBs where AREG was expressed, while AREG expression was rare in hyperplastic and tumor structures in which PyMT staining was present (Fig. 3a, PyMT panel).

In both AREG^+/+^ PyMT and AREG^−/−^ PyMT mammary glands, all lesions express PyMT (Fig. 3b). Interestingly, the intensity of PyMT fluorescence in these lesions is similar in AREG^+/+^ PyMT and AREG^−/−^ PyMT mammary glands (Fig. 3c). Since the lesions are larger in AREG^−/−^ PyMT MFPs, this suggests that PyMT is expressed in more areas in AREG^−/−^ PyMT mammary glands. However, the level of expression does not differ in the absence of AREG.

### Absence of AREG results in reduced myoepithelial cell number, coverage, and thickness

**Fig. 4:**
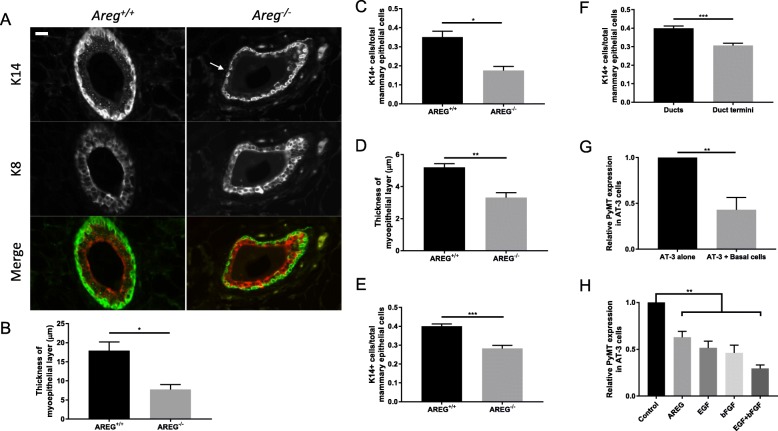
Myoepithelial layer has fewer cells and is thinner in thickness in AREG^−/−^ mice. **a** Representative images of 12-week-old AREG^+/+^ (left) and AREG^−/−^ (right) mammary ducts immunostained with K8 (red) and K14 (green) in merged channel. Arrow indicates discontinuous myoepithelial layer. Scale bar shows 50 μm. At both 6 weeks (**b, c**) and 12 weeks (**d, e**), AREG^−/−^ glands have thinner myoepithelial layer (**b, d**) and smaller percentage of K14^+^ cells (**c, e**). At least three animals used in each analysis. **f** Proportion of K14^+^ cells in ducts and mature duct termini of 12-week-old AREG^+/+^ mammary ducts compared (*N* = 7). **g** PyMT expression in AT-3 cells suppressed by myoepithelial cells when cocultured. AT-3 cells either cultured alone or with primary myoepithelial cells overnight. RNA extracted and PyMT expression assessed by RT-qPCR. **h** AT-3 cells cultured without addition of growth factors (control), with 100 ng/ml AREG, 10 ng/ml EGF, or 10 ng/ml bFGF, or with both EGF and bFGF. Statistical analysis performed using *t* test. *p < 0.05, **p < 0.01, ***p < 0.001. AREG amphiregulin, bFGF basic fibroblast growth factor, EGF epidermal growth factor, PYMT polyoma middle-T antigen

Even though the ductal tree is much smaller than in the wildtype, lesion growth in AREG^−/−^ PyMT mice was significantly greater. We examined the mammary ducts of 6-week-old and 12-week-old AREG^+/+^ and AREG^−/−^ mice in the absence of PyMT to determine whether differences in the mammary ducts may explain the enhanced lesion growth in AREG^−/−^ mice. We used cytokeratin-8 (K8) and cytokeratin-14 (K14) staining to visualize the luminal and myoepithelial layers of the mammary duct, respectively. The myoepithelial cell layer in the AREG^−/−^ mice was often discontinuous (Fig. 4a, arrow) and K14 staining cells could be seen also in the luminal layer. Overall, the myoepithelial cell layer was thinner (Fig. 4b, d) and the proportion of K14^+^ myoepithelial cells was smaller (Fig. 4c, e) in both 6-week-old and 12-week-old AREG^−/−^ mice. Since it is possible that PyMT tumors initiate from mature duct termini in AREG^+/+^ mice, we compared the proportion of myoepithelial cells in ducts and terminal acini in AREG^+/+^ mammary glands and found that there were fewer myoepithelial cells in the acini as well (Fig. 4f).

Because myoepithelial cells are recognized to be tumor suppressors [29], we hypothesized that myoepithelial cells might be able to suppress PyMT expression. A reduction in myoepithelial cells in the AREG^−/−^ mammary ducts might lead to induction of PyMT expression in more ductal cells, resulting in more lesion formation in AREG^−/−^ PyMT mice. To test this hypothesis, we cocultured primary myoepithelial cells with AT-3 cells, a breast tumor cell line derived from C57Bl/6 MMTV-PyMT mammary tumors [30]. When cocultured with primary myoepithelial cells, PyMT expression in AT-3 cells was significantly reduced (Fig. 4g).

In addition, AREG and FGFR signaling are critical for proper mammary gland elongation and branching [31]. We therefore examined PyMT expression in AT-3 cells cultured in the presence of the EGFR ligands EGF and AREG, as well as FGFR ligand bFGF. In the presence of AREG, EGF, or bFGF, PyMT expression in AT-3 cells was reduced (Fig. 4h). Therefore, a loss of AREG expression in vivo could contribute to the broader expression of PyMT seen in the AREG^−/−^ mice through both a reduction in myoepithelial cells as well as reduced EGFR and FGFR signaling.

### Late-stage AREG^−/−^ tumors are histologically distinct from AREG^+/+^ tumors

**Fig. 5:**
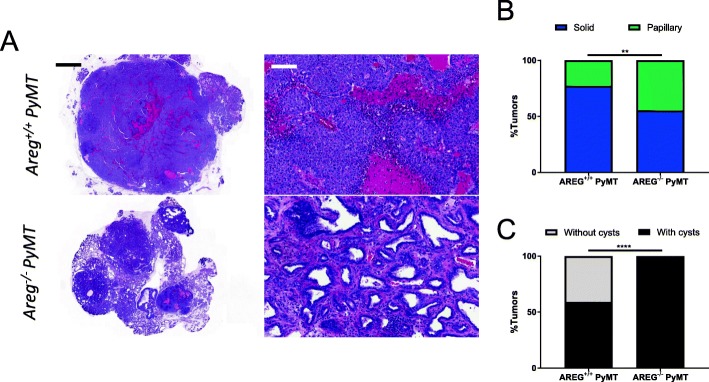
Late-stage AREG^−/−^ tumors less solid with greater proportion of papillary tumor features. **a** One-centimeter tumors from AREG^+/+^ PyMT (*N* = 32) and AREG^−/−^ PyMT (*N* = 22) mice stained with H&E. Scale bars for whole tumors and sections 2000 μm (left) and 100 μm (right), respectively. b Proportion of solid and papillary tumor areas determined for each tumor. **c** Proportion of AREG^+/+^ PyMT (*N* = 32) and AREG^−/−^ PyMT (*N* = 22) tumors that have cysts or no cysts. Statistical analysis performed using Mann–Whitney test (**b**) and chi-square test (**c**). **p < 0.01, ****p < 0.0001. AREG amphiregulin, PYMT polyoma middle-T antigen

Late-stage (1-cm diameter as measured by caliper) AREG^+/+^ PyMT tumors are characterized by solid sheets of cells with occasional ductal structures remaining at the periphery [15]. However, size-matched (1-cm diameter) AREG^−/−^ PyMT tumors are more heterogeneous in their histology; they are often composed of both solid and papillary tumor areas (Fig. 5a). These papillary tumor regions are characterized by finger-like fronds that are composed of fibrovascular stalks lined by neoplastic epithelial cells [32]. When we compared the percentage of each tumor occupied by solid or papillary histology, we found that AREG^−/−^ tumors had a significantly greater proportion of papillary tumor histology than AREG^+/+^ tumors (Fig. 5b). We also noticed that many tumors, particularly AREG^−/−^ tumors, are very cystic (Additional file 3: Figure S3). If a tumor had at least one cyst, we characterized that tumor as cystic. Our analysis revealed that while some AREG^+/+^ tumors are cystic, all of the AREG^−/−^ tumors had cysts (Fig. 5c). Thus, in addition to having more papillary features, the AREG^−/−^ tumors are also more cystic.

**Fig. 6:**
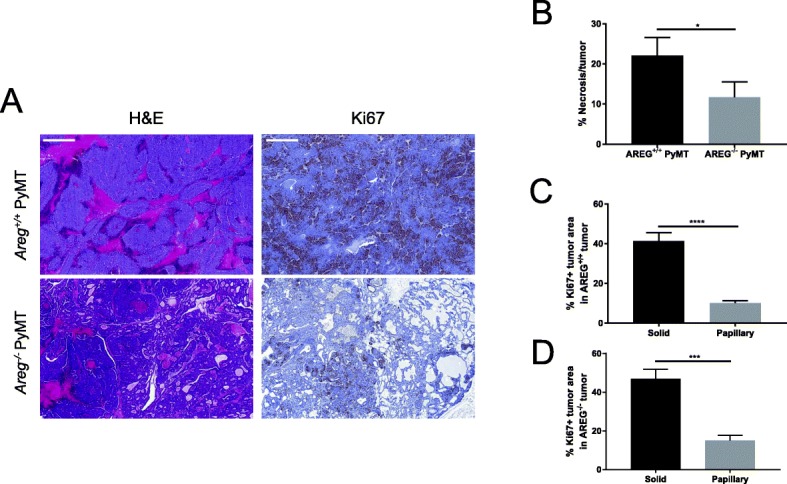
AREG^−/−^ tumors are less necrotic and tumor cells in papillary regions are less proliferative. **a** Representative images of H&E (left column) and Ki67 (right column) staining in AREG^+/+^ PyMT and AREG^−/−^ PyMT 1-cm tumors. Scale bar shows 500 μm for H&E stains and Ki67 stains. **b** Percentage of necrotic areas calculated as average of five fields per AREG^+/+^ (*N* = 32) and AREG^−/−^ (*N* = 22) 1-cm tumors. **c, d** Ki67^+^ proliferating cells in solid vs papillary areas in 1-cm AREG^+/+^ PyMT and AREG^−/−^ PyMT tumors compared using HistoQuant. Evaluation performed on at least five separate areas from at least three different tumors per genotype. Statistical analysis performed using *t* test. *p < 0.05, ***p < 0.001, ****p < 0.0001. AREG amphiregulin, H&E hematoxylin and eosin, PYMT polyoma middle-T antigen

To better understand the implications of histological differences on tumor growth, we assessed AREG^+/+^ and AREG^−/−^ tumors for the presence of necrosis (Fig. 6a, H&E images). In both types of tumors, there is considerable variability in the amount of necrosis, ranging from none at all to over 50% necrotic. Overall, AREG^−/−^ tumors are less necrotic than the wildtype counterpart (Fig. 6b). When we compared necrotic areas between solid areas of AREG^+/+^ tumors and AREG^−/−^ tumors, a significant difference remains while the papillary areas have little to no necrosis regardless of AREG status (Additional file 4: Figure S4). This suggests that the differences in necrosis we have observed between AREG^+/+^ and AREG^−/−^ tumors are due to both an increased proportion of papillary tumor histology (which is not necrotic) as well as reduced necrosis in the solid tumor regions in the AREG^−/−^ tumors.

We also immunostained these tissues for Ki67 (Fig. 6a, Ki67 images). We compared the Ki67^+^ areas in solid and papillary regions and found that in both AREG^+/+^ and AREG^−/−^ tumors, papillary regions have less Ki67 staining (Fig. 6c, d). Since AREG^+/+^ tumors are proportionally more solid than AREG^−/−^ tumors, this indicates that, as a whole, AREG^+/+^ tumors are more proliferative than AREG^−/−^ tumors. However, this may be counteracted by increased necrosis: in the solid areas where tumor proliferation is high, tumor cells far from the blood vessel do not receive enough oxygen and nutrients and become hypoxic [33–35]. Rapid growth of the tumor causes exhaustion of the nutrients and oxygen supplied by the nearby blood vessels and, as a result, forms necrotic zones. Conversely, in the papillary and cystic areas that are more common in the AREG^−/−^ tumors, there is slower growth, more stroma, and correspondingly less necrosis.

We used CD31 staining to compare the vasculature between AREG^+/+^ and AREG^−/−^ tumors (Additional file 5: Figure S5A). The vessel structures in AREG^+/+^ tumors are thin and long while the vessels in AREG^−/−^ tumors appear shorter and irregular in shape. These observations are complemented by quantification of CD31 signals, showing increased numbers of CD31^+^ vessels in AREG^−/−^ tumors (Additional file 5: Figure S5B).

### Lower AREG expression is associated with papillary breast cancer

**Fig. 7:**
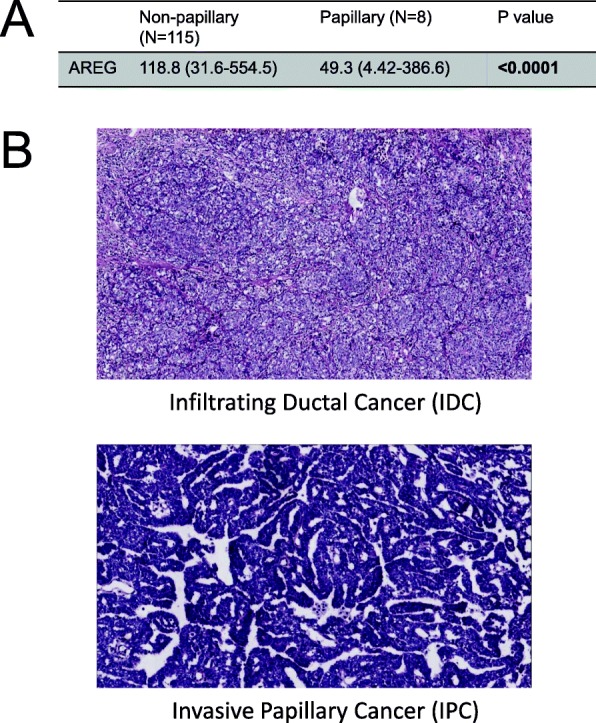
Lower AREG expression associated with papillary breast cancer. **a** AREG expression compared between 115 luminal-B nonpapillary IDC samples and eight luminal-B IPC samples. **b** Representative H&E images of luminal-B IDC and luminal-B IPC. Statistical analysis performed using Mann–Whitney test. *p* < 0.0001. AREG amphiregulin

From our results using the PyMT mouse model, we found that the absence of AREG changes the tumor histological growth pattern, with AREG^−/−^ tumors developing with more papillary features. In human breast cancer, invasive papillary cancer (IPC) is a subtype of infiltrating ductal carcinoma (IDC). The genomic profile of the PyMT mouse model has been characterized as most similar to the luminal B molecular subtype [36]. Therefore, we examined The Cancer Genome Atlas (TCGA) for patient samples that have been identified as luminal B. Using the available pathological reports, the patient samples were separated into nonpapillary IDC versus IPC. We then evaluated the AREG expression of these tumors as provided in TCGA. Interestingly, we found that patients with IPC have a significantly lower AREG expression than those with nonpapillary IDC (Fig. 7), consistent with our results that AREG^−/−^ tumors are more papillary.

## Discussion

In our studies, we found that the loss of AREG resulted in accelerated expansion of PyMT-positive lesions in the PyMT mouse model of breast cancer. As early as 6 weeks of age, lesions in AREG^−/−^ PyMT mice were increasing in size more quickly. Lesions in both AREG^+/+^ PyMT mice and AREG^−/−^ PyMT mice were PyMT-positive. However, it was unclear why there was a larger PyMT-positive area in the AREG^−/−^ PyMT mice. When we compared the cellular composition of the mammary ducts in AREG^+/+^ and AREG^−/−^ pubertal and adult mice, we found that AREG^−/−^ ducts had fewer myoepithelial cells and thinner myoepithelial cell layers than AREG^+/+^ ducts. Interestingly, in AREG^+/+^ PyMT MFPs, the PyMT-positive lesions do not express AREG. We cocultured primary myoepithelial cells with AT-3 cells, a breast tumor cell line derived from the MMTV-PyMT mouse model, and found a significant reduction in PyMT expression in the AT-3 cells. Furthermore, when we cultured AT-3 cells with EGFR ligands AREG and EGF, or FGFR ligand bFGF, PyMT expression was reduced. In late-stage AREG^−/−^ tumors, we also found a striking difference in the tumor growth pattern. Most of the AREG^−/−^ tumors presented with increased papillary histology, cysts, and number of intratumoral vessels. Finally, we compared the tumor histology and AREG expression of luminal B breast cancer patient samples in TCGA and found papillary breast cancer was associated with low AREG expression.

In the PyMT model, PyMT expression in the mammary gland is driven by the MMTV promoter [37]. Stimulation of the MMTV promoter is primarily controlled by binding of glucocorticoid-bound glucocorticoid receptor complexes to the hormone receptor element in the long terminal repeat (LTR) region of the MMTV promoter [38, 39]. Interestingly, EGF has been shown to stimulate tyrosine phosphorylation of the glucocorticoid receptor in breast epithelial HBL100 cells [40]. As a result of EGF stimulation, binding of dexamethasone to the glucocorticoid receptor is reduced [41]. Dexamethasone treatment inhibits proliferation of HBL100 cells. However, adding EGF to the dexamethasone treatment overcomes dexamethasone-mediated inhibition of cell proliferation. EGF has also been shown to increase the expression of *Egr2*, a gene that is inhibited by glucocorticoids [42, 43]. This suggests that activation of the EGFR signaling pathway can reduce GR signaling, which is important for MMTV promoter stimulation. Thus, EGF, and possibly AREG, may suppress PyMT expression through inhibition of glucocorticoids binding to its receptor.

It is also possible that loss of AREG alters the balance of proliferation between different cell types that can contribute to formation of a tumor. AREG expression might suppress the proliferation of cells with the capability of driving MMTV promoter activity, and then loss of AREG could lead to increased proliferation of such cells. Alternatively, AREG may bias differentiation. The cell fate of mammary epithelial progenitors has been shown to be partially dependent on EGFR signaling during development [14]. Under high levels of EGFR activation, progenitor cells preferentially differentiate into luminal epithelial cells. The common luminal progenitor gives rise to both ER-positive and ER-negative ductal cells as well as ER-negative alveolar cells [44]. Potentially, the loss of AREG could bias differentiation toward the alveolar cell phenotype, resulting in more cells that can express PyMT. Further studies will be needed to resolve these possibilities.

Furthermore, we have provided evidence that myoepithelial cells can also reduce PyMT expression. Although the mechanism is unknown, myoepithelial cells have a plethora of activities and functions, aside from the canonical mechanical contractile function. In particular, it has been shown that myoepithelial cells are tumor suppressors, involved in the inhibition of breast tumor cell proliferation in vitro and breast tumor growth in vivo, as well as angiogenesis [45, 46]. Myoepithelial cells also produce activin, a member of the TGF-β superfamily, which can also inhibit breast cancer cell proliferation by activating cell cycle arrest mediated by Smads [47, 48]. In other studies, TGF-β negatively regulates MMTV expression in a mammary tumor cell line [49]. Therefore, it is possible that myoepithelial cell-secreted factors may reduce PyMT expression via suppression of the MMTV promoter.

**Fig. 8:**
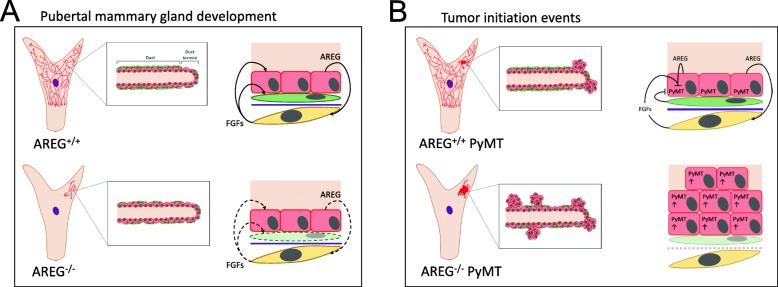
Working model to explain increased tumor initiation and morphological changes in AREG^−/−^ tumors. (**a**) In mammary duct in absence of PyMT (top), myoepithelial cells (green) form continuous layer around luminal epithelial cells. In mature duct termini, myoepithelial layer is discontinuous. Luminal epithelial cells secrete AREG that binds to EGFR on stromal cells (yellow). Stimulated stromal cells produce FGFR ligands that bind to FGFR luminal epithelial cells and myoepithelial cells [31, 56]. In absence of AREG (bottom), EGFR/FGFR paracrine loop is interrupted and impairs proper mammary ductal development. (**b**) In AREG^+/+^ PyMT animals (top), PyMT initiates transformation of luminal epithelial cells in mature duct termini where there are fewer myoepithelial cells. Myoepithelial cells as well as secreted growth factors such as AREG and bFGF suppress PyMT expression in mammary duct. In AREG^−/−^ PyMT mice (bottom), PyMT expression is more widespread. Due to global reduction in myoepithelial cells and reduced AREG and FGF expression, oncogenic transformation takes place more broadly in ductal tree. AREG amphiregulin, FGF basic fibroblast growth factor, PYMT polyoma middle-T antigen

From our studies, we have developed a model that summarizes our key findings (Fig. 8). As key mediators of estrogen-induced mammary ductal development, epithelial AREG and stromal EGFR promote ductal elongation during puberty [11, 13]. Paracrine EGFR–FGFR signaling between mammary epithelial and neighboring stromal cells has been shown to be critical in proper ductal growth and branching [31]. In AREG^+/+^ mice (Fig. 8a, top), AREG stimulates fibroblasts to produce FGFs, which bind to FGF receptors on the epithelial cells to stimulate ductal growth and branching. In AREG^−/−^ mice (Fig. 8a, bottom), there is diminished paracrine signaling between mammary epithelial cells and stromal cells, and as a result the ductal tree fails to grow beyond the postnatal stage and myoepithelial cell coverage is reduced in the ducts. In the AREG^+/+^ PyMT mice (Fig. 8b, top), PyMT expression may be suppressed in ducts and terminal end buds both by myoepithelial cells and growth factors such as AREG, EGF, and bFGF, or there is suppression of the generation of cells that can expression PyMT. We propose that in AREG^+/+^ PyMT mice, tumors are initiated at mature duct termini where myoepithelial coverage is lower, giving rise to the observed multifocal initiation. On the other hand, in the AREG^−/−^ PyMT mice, both myoepithelial coverage and growth factor expression are reduced, leading to broader PyMT expression resulting in increased lesion formation throughout the ductal tree. As a result, AREG^−/−^ tumors form a wider range of tumor morphologies, including less aggressive papillary and cystic structures.

Currently, treatment of breast cancer with receptor tyrosine kinase (RTK) inhibitors that target EGFR, such as gefitinib, has been met with mixed success [4–6]. Getfitnib treatment in patients with hormone receptor-positive or hormone receptor-negative metastatic breast cancer is associated with low clinical benefit rate (CBR). These treatments are also commonly associated with cutaneous, gastrointestinal, and hair-related toxicities [50, 51]. Therefore, EGFR-targeted therapies are not well tolerated and have low efficacy for some patients. AREG as a novel target for breast cancer therapy is attractive [52, 53], potentially reducing the side effects of broad EGFR inhibition while targeting breast tumor growth that is driven by AREG. One study using an antibody against AREG in ovarian cancer xenografts has successfully reduced tumor growth [54]. In our model, loss of AREG dramatically altered the histological morphology seen in late-stage tumors from a solid to a papillary structure. In human breast cancer, papillary carcinoma is associated with a higher survival rate than invasive ductal carcinoma [55]. AREG-targeted therapy could avoid negative side effects associated with broad EGFR inhibitors, but could also potentially direct tumor growth toward a less aggressive pattern.

## Conclusions

Our studies demonstrate a novel role of AREG in myoepithelial cell coverage of mammary ducts during development. In the PyMT model of breast cancer, we have shown that myoepithelial cells and growth factors AREG, EGF, and bFGF suppress PyMT expression. These findings may explain the accelerated growth and progression of early-stage AREG^−/−^ tumors in the MMTV-PyMT model. Interestingly, late-stage AREG^−/−^ tumors are less proliferative and demonstrate increased areas of papillary and cystic features. In human breast cancer, luminal-B IPCs have lower AREG expression compared to IDCs. Together, our results provide new insight into the function of AREG in mammary gland biology, regulation of PyMT, and breast tumor growth.

## Additional files

Additional file 1: **Figure S1** Growth of AREG^+/+^ PyMT and AREG^−/−^ PyMT lesions. Volumes of palpable lesions that could be reproducibly detected in AREG^+/+^ PyMT (**A**, *N* = 32) and AREG^−/−^ PyMT (**B**, *N* = 22) mice were measured using a digital caliper. (**C**) Kaplan–Meier plot of percentage of mice with no palpable lesions. Statistical analysis performed using a log-rank test (PPTX 143 kb)

Additional file 2: **Figure S2** Loss of AREG does not have a significant effect on tumor cell intravasation and metastasis. (**A**) Number of metastatic foci in lungs of AREG^+/+^ PyMT (*N* = 16) and AREG^−/−^ PyMT (*N* = 9) mice. (**B**) Total area of all metastatic foci in each lung calculated for AREG^+/+^ PyMT (*N* = 16) and AREG^−/−^ PyMT (*N* = 9) mice. (**C**) Blood collected from right atrium of AREG^+/+^ PyMT (*N* = 8) and AREG^−/−^ PyMT (*N* = 8) mice, and CTCs counted and number adjusted to 1 ml of blood. Statistical analysis performed using Mann–Whitney test. n.s. not significant (PPTX 124 kb)

Additional file 3: **Figure S3** Cysts present in AREG^+/+^ PyMT and AREG^−/−^ PyMT tumors. H&E stains of AREG^+/+^ PyMT and AREG^−/−^ PyMT 1-cm tumors show presence of cysts. Scale bar shows 2000 μm (PPTX 2060 kb)

Additional file 4: **Figure S4** Necrosis reduced in solid areas of AREG^−/−^ PyMT tumors. Percentage necrosis in solid and papillary areas of AREG^+/+^ PyMT (*N* = 32) and AREG^−/−^ PyMT (*N* = 22) tumors assessed individually. Significant differences observed between solid areas of AREG^+/+^ PyMT and AREG^−/−^ PyMT tumors. In addition, papillary regions of both tumor genotypes have little to no necrosis. Statistical analysis performed using Mann–Whitney test. *p < 0.05, ***p < 0.001, ****P < 0.0001. n.s. not significant (PPTX 61 kb)

Additional file 5: **Figure S5** Loss of AREG is associated with increased vascular density in late-stage mammary tumors. (**A**) Representative images of CD31 staining of AREG^+/+^ PyMT and AREG^−/−^ PyMT 1-cm tumors. (**B**) Compared to AREG^+/+^ PyMT tumors, more CD31^+^ vessels per field in AREG^−/−^ PyMT tumors. Scale bar shows 100 μm. Statistical analyses performed using a *t* test. *p < 0.05, *N* = 3 (PPTX 182 kb)
