# Supplementary figures and images for: Loss of amphiregulin reduces myoepithelial cell coverage of mammary ducts and alters breast tumor growth
Source: Breast Cancer Res. 2018 Oct 26;20:131. doi: 10.1186/s13058-018-1057-0 (PMC6203982; doi:10.1186/s13058-018-1057-0)

## Slide 1
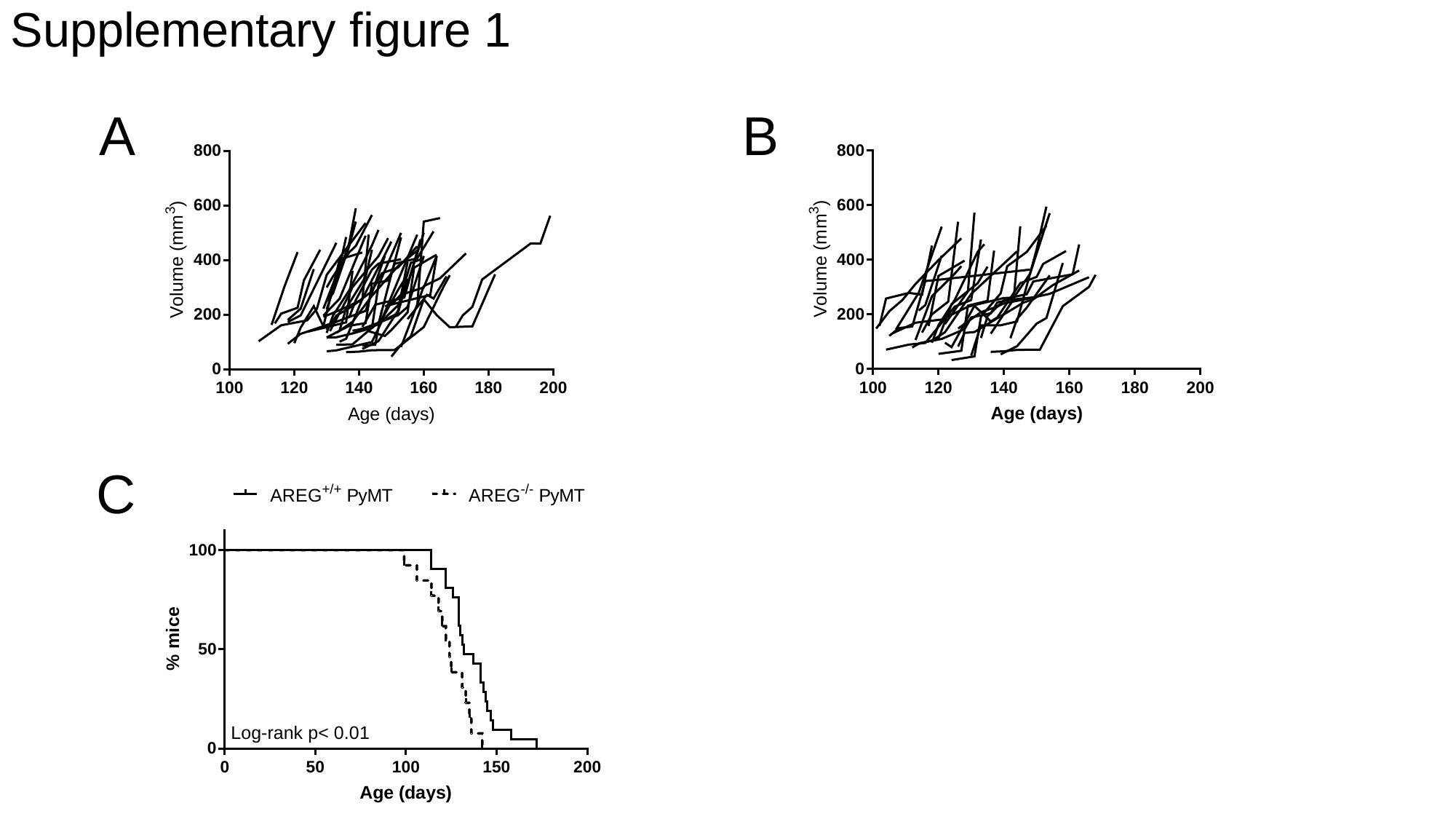

Supplementary figure 1
A
B
C

Supplement: Supplementary file 1 — Figure S1 Growth of AREG+/+ PyMT and AREG−/− PyMT lesions. Volumes of palpable lesions that could be reproducibly detected in AREG+/+ PyMT (A, N = 32) and AREG−/− PyMT (B, N = 22) mice were measured using a digital caliper. (C) Kaplan–Meier plot of percentage of mice with no palpable lesions. Statistical analysis performed using a log-rank test (PPTX 143 kb) [file 13058_2018_1057_MOESM1_ESM.pptx]

## Slide 1
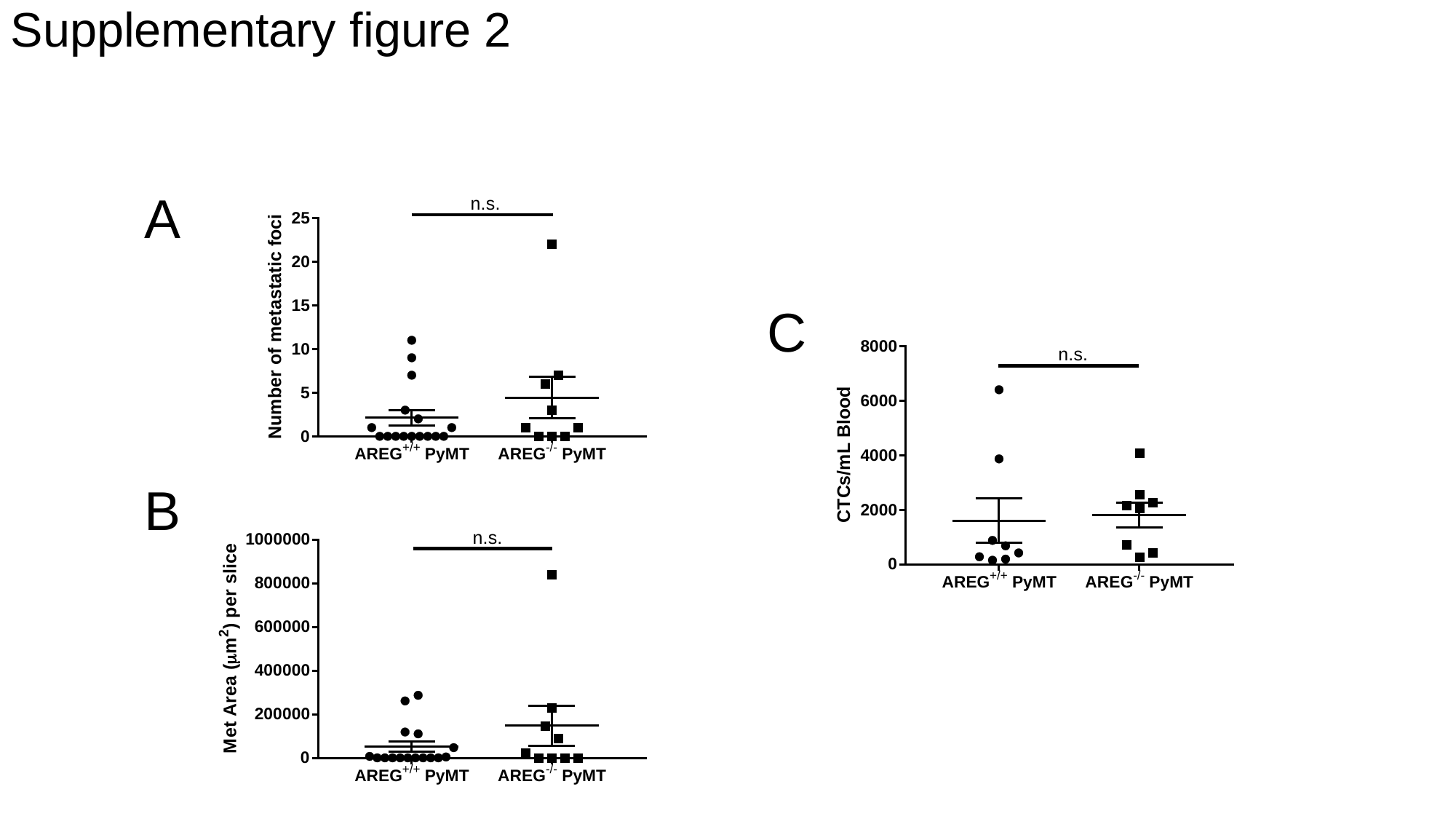

Supplementary figure 2
A
C
B

Supplement: Supplementary file 2 — Figure S2 Loss of AREG does not have a significant effect on tumor cell intravasation and metastasis. (A) Number of metastatic foci in lungs of AREG+/+ PyMT (N = 16) and AREG−/− PyMT (N = 9) mice. (B) Total area of all metastatic foci in each lung calculated for AREG+/+ PyMT (N = 16) and AREG−/− PyMT (N = 9) mice. (C) Blood collected from right atrium of AREG+/+ PyMT (N = 8) and AREG−/− PyMT (N = 8) mice, and CTCs counted and number adjusted to 1 ml of blood. Statistical analysis performed using Mann–Whitney test. n.s. not significant (PPTX 124 kb) [file 13058_2018_1057_MOESM2_ESM.pptx]

## Slide 1
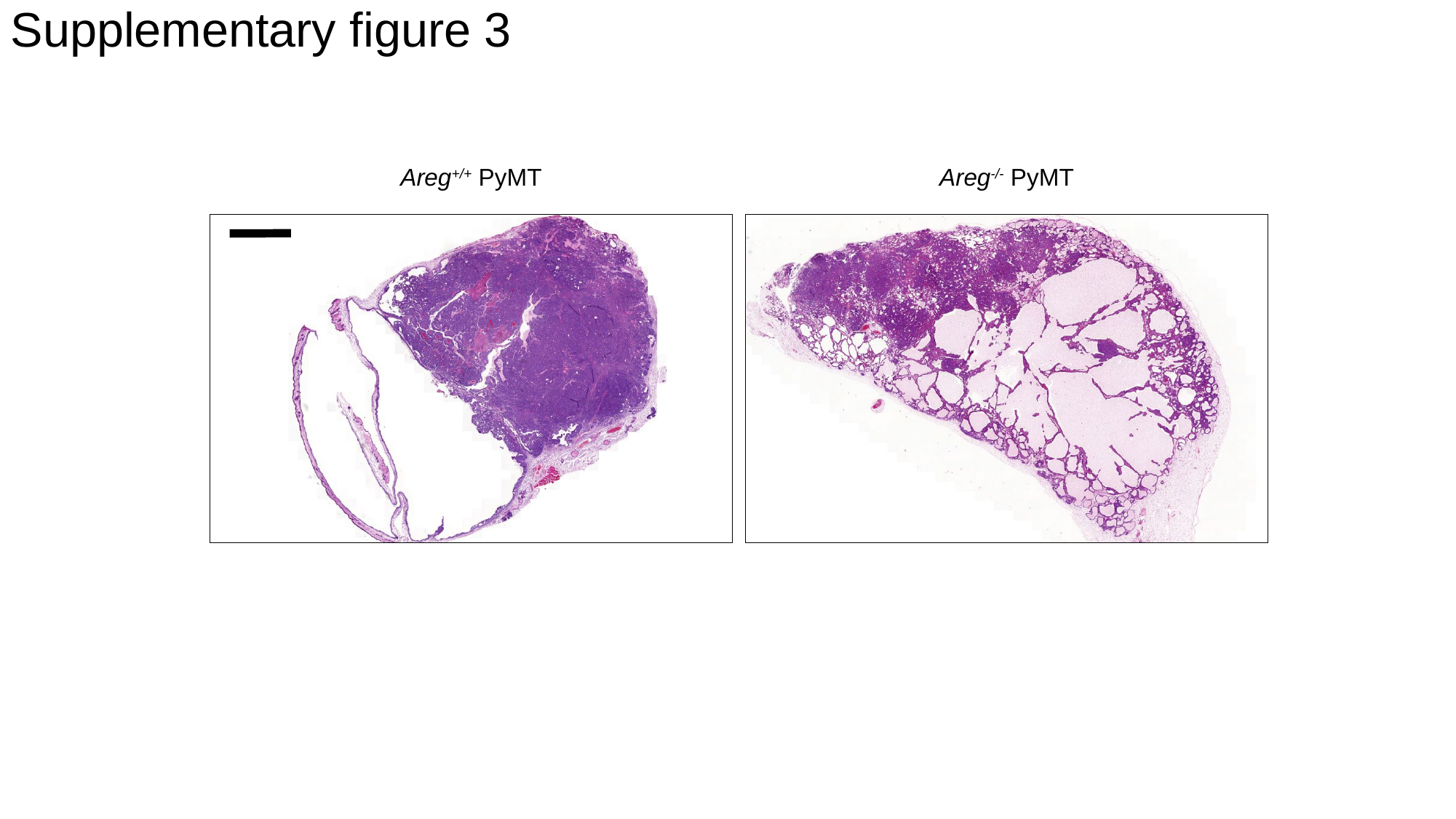

Supplementary figure 3
Areg+/+ PyMT
Areg-/- PyMT

Supplement: Supplementary file 3 — Figure S3 Cysts present in AREG+/+ PyMT and AREG−/− PyMT tumors. H&E stains of AREG+/+ PyMT and AREG−/− PyMT 1-cm tumors show presence of cysts. Scale bar shows 2000 μm (PPTX 2060 kb) [file 13058_2018_1057_MOESM3_ESM.pptx]

## Slide 1
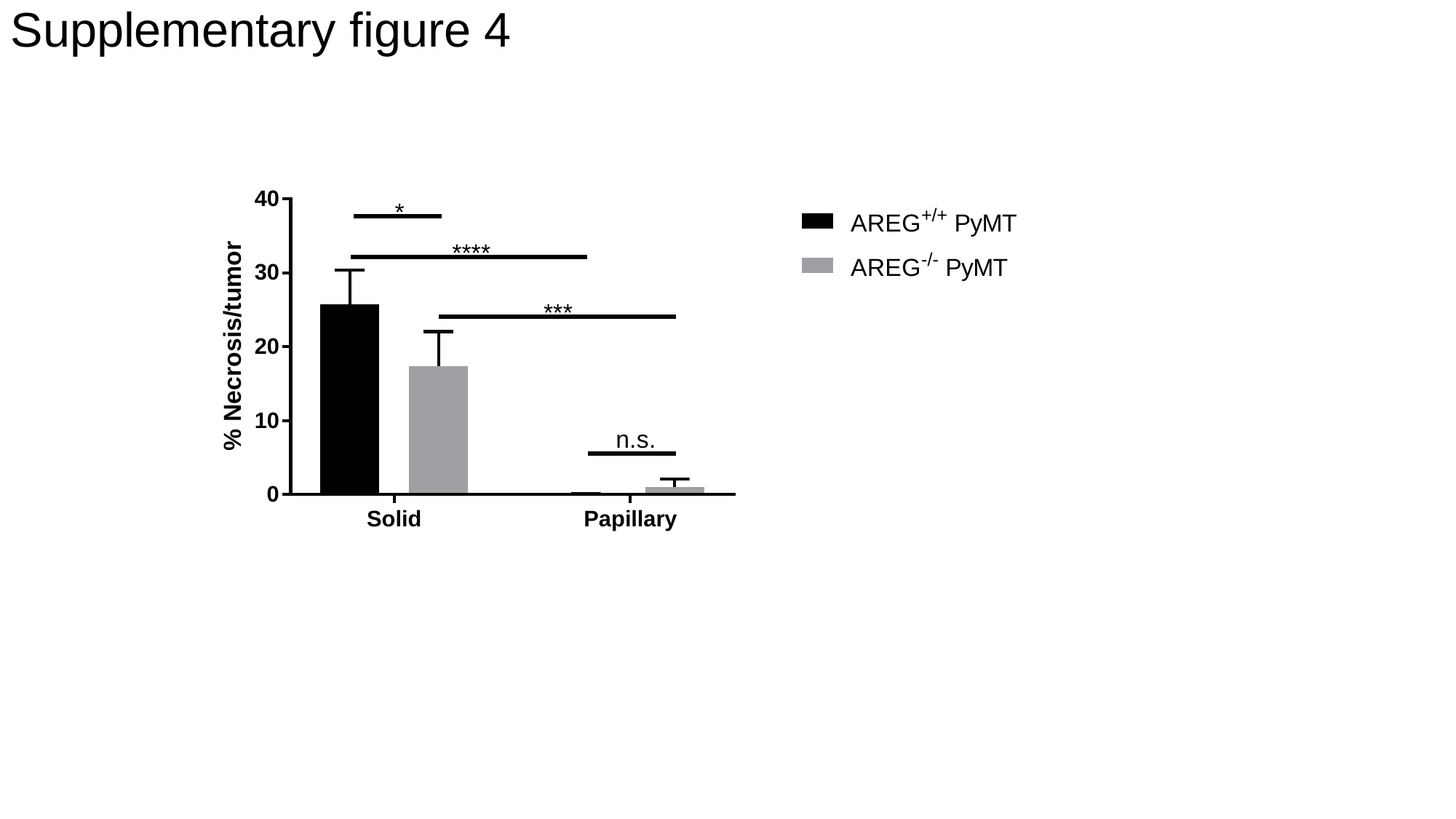

Supplementary figure 4

Supplement: Supplementary file 4 — Figure S4 Necrosis reduced in solid areas of AREG−/− PyMT tumors. Percentage necrosis in solid and papillary areas of AREG+/+ PyMT (N = 32) and AREG−/− PyMT (N = 22) tumors assessed individually. Significant differences observed between solid areas of AREG+/+ PyMT and AREG−/− PyMT tumors. In addition, papillary regions of both tumor genotypes have little to no necrosis. Statistical analysis performed using Mann–Whitney test. *p < 0.05, ***p < 0.001, ****P < 0.0001. n.s. not significant (PPTX 61 kb) [file 13058_2018_1057_MOESM4_ESM.pptx]

## Slide 1
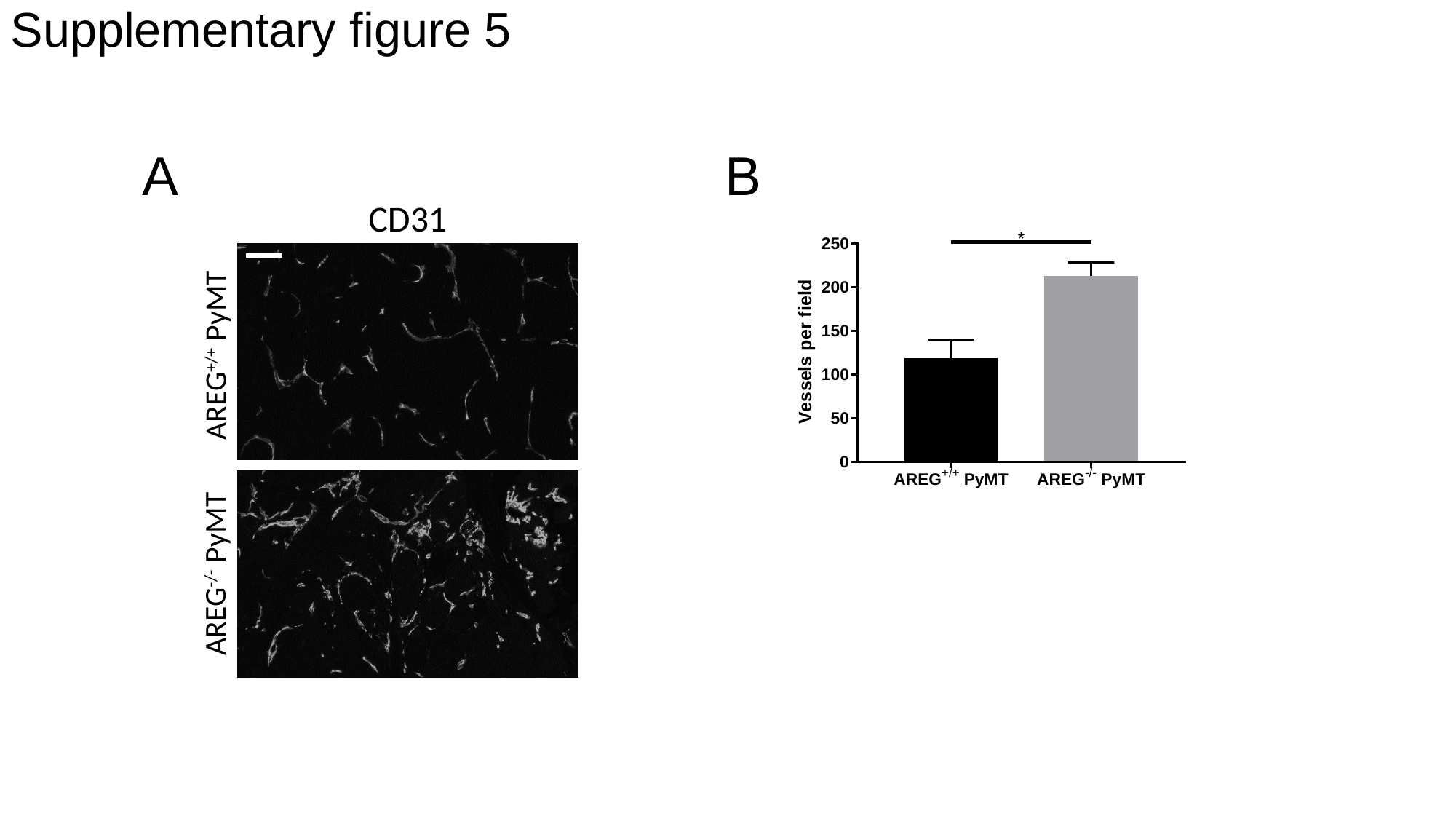

Supplementary figure 5
A
B
CD31
AREG+/+ PyMT
AREG-/- PyMT

Supplement: Supplementary file 5 — Figure S5 Loss of AREG is associated with increased vascular density in late-stage mammary tumors. (A) Representative images of CD31 staining of AREG+/+ PyMT and AREG−/− PyMT 1-cm tumors. (B) Compared to AREG+/+ PyMT tumors, more CD31+ vessels per field in AREG−/− PyMT tumors. Scale bar shows 100 μm. Statistical analyses performed using a t test. *p < 0.05, N = 3 (PPTX 182 kb) [file 13058_2018_1057_MOESM5_ESM.pptx]
